# Temperature Controlled Loading and Release of the Anti-Inflammatory Drug Cannabidiol by Smart Microgels

**DOI:** 10.3390/molecules26113181

**Published:** 2021-05-26

**Authors:** Maxim Dirksen, Timo Alexander Kinder, Timo Brändel, Thomas Hellweg

**Affiliations:** 1 Physical and Biophysical Chemistry, Bielefeld University, Universitätsstraße 25, 33615 Bielefeld, Germany; dirksen@uni-bielefeld.de (M.D.); timo.braendel@daikinchem.de (T.B.); 2 Cannovum AG, Rheinsberger Str. 76/77, 10115 Berlin, Germany; timo.kinder@cannovum.com; 3 Daikin Chemical Europe GmbH, Am Wehrhahn 50, 40211 Düsseldorf, Germany

**Keywords:** drug delivery, thermoresponsive microgels, CBD, NIPAM, NIPMAM, natural drugs

## Abstract

CBD is a promising candidate for treatment of many diseases and plays a major role in the growing trend to produce high-end drugs from natural, renewable resources. In the present work, we demonstrate a way to incorporate the anti-inflammatory drug CBD into smart microgel particles. The copolymer microgels that we chose as carrier systems exhibit a volume phase transition temperature of 39 ${}^{\circ }C$, which is just above normal body temperature and makes them ideal candidates for hyperthermia treatment. While a simple loading route of CBD was not successful due to the enormous hydrophobicity of CBD, an alternative route was developed by immersing the microgels in ethanol. Despite the expected loss of thermoresponsive behaviour of the microgel matrix due to the solvent exchange, a temperature-dependent release of CBD was detected by the material, creating an interesting question of interactions between CBD and the microgel particles in ethanol. Furthermore, the method developed for loading of the microgel particles with CBD in ethanol was further improved by a subsequent transfer of the loaded particles into water, which proves to be an even more promising approach due to the successful temperature-dependent release of the drug above the collapse temperature of the microgels.

## 1. Introduction

Individual concepts of drug delivery and targeting mechanisms play a key role in future developments in pharmacy, with many new drugs emerging in the pharmaceutical market in the last few decades. Acrylamide-based responsive microgels have been discussed as potential candidates for this in several publications, including two recent reviews [1,2]. Generally, microgels are described as three-dimensional polymer networks, swollen by a solvent and exhibiting a size in the colloidal range [3]. Their versatility and adaptability make microgels outstanding candidates for many applications beyond drug delivery, including oil recovery [4], emulsifiers [5], nanoparticle carriers [6,7,8,9] and cell culture substrates [10].

**Figure 1:**
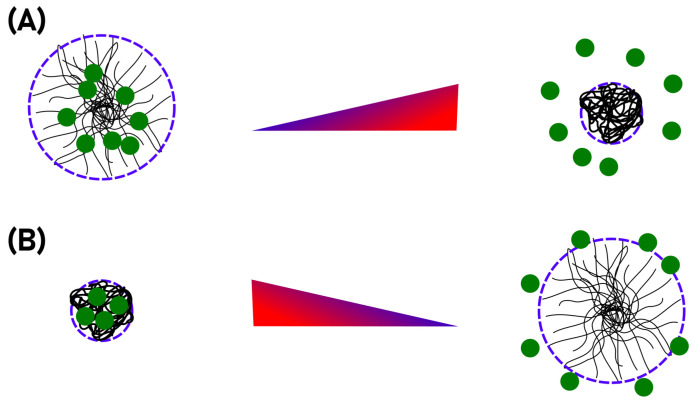
Two main concepts of microgel usage as drug carrier. In a hyperthermia treatment, the drug is loaded into the swollen gel and upon temperature increase it is pressed out of the polymer network alongside the solvent (**A**). Extremely hydrophobic substances may also be loaded in the collapsed state and released from the network into an hydrophobic environment upon swelling connected to a temperature decrease (**B**).

One of the most remarkable properties of microgels is their ability to respond to different external stimuli, e.g., temperature, pH-value and ionic strength. Our study focuses on the thermoresponsive materials poly(*N*-isopropylacrylamide) (PNIPAM) and poly(*N*-isopropylmethacrylamide) (PNIPMAM). At a certain temperature, the so-called volume phase transition temperature (VPTT), the particles undergo a drastic decrease in size. In contrast to linear PNIPAM chains, which precipitate above the LCST of ca. 32 ${}^{\circ }C$ [11], PNIPAM microgels maintain their colloidal stability due to their synthesis-related surface charges. Moreover, it is very advantageous that the properties of microgels can be tailored precisely, for instance via the variation of the monomer composition [12,13,14], addition of surfactants [15] and by the realization of specific particle architectures [16,17]. Just to give two examples, it is easily possible to generate core-shell architectures [18,19] or hollow spheres [20]. However, while tailoring microgels is of great importance when it comes to specific applications, the general scheme of drug delivery always makes use of the very basic volume phase transition behaviour of the particles. Depending on the properties of the drug which is loaded, two scenarios are imaginable (see Figure 1). The mechanism depicted in Figure 1A is usually employed in the context of hyperthermia treatments where the dug is loaded into the swollen polymer network. The drug is only released when the VPTT is crossed. This way, the delivery can be triggered easily by heating certain parts of the body above the VPTT. Obviously, it is necessary to tune the VPTT of the microgels to be higher than the body temperature, but this can be achieved easily by copolymerization, as described by Djopke and Vogt [21] and Wedel et al. [22]. Another possibility for the loading and release of a drug is shown in Figure 1B. Here, the loading takes place in the collapsed state of the carrier system. An increase in temperature leads to the swelling of the particles, so that the active ingredient is released [16,23].

Regarding the use of microgels as drug-delivery vehicles, many different studies have been performed with varying drugs, ranging from lysozyme [24] over the more hydrophilic drugs dopamine, naproxen, desipramine, dibucaine, acetaminophen [25], and β-aescin [26] to the hydrophobic doxorubicin (DOX) [27,28,29,30,31,32,33]. Just from these representative examples, it can be stated that the structure of microgels, which often exhibit a rather hydrophobic, cross-linker rich core region and a rather hydrophilic corona [34], enables these particles to act as carriers for a variety of hydrophilic and hydrophobic drugs. In particular, very hydrophobic drugs often lead to problems with commercial carrier systems, due to their non-existent solubility in water. A possible approach for the delivery of such hydrophobic drugs was presented by Kataoka et al. [35] These authors used block copolymer micelles consisting of PEO-PBLA and loaded these systems with DOX by an o/w emulsion method. In this context, a good loading is achieved, and the drug is released slowly from the carrier system without the active ingredient being adversely affected. Compared to Kataokas approach, microgels could potentially be advantageous for such an application, as their ability to react to external stimuli such as temperature [26] or pH [36] can be easily utilized to realize a targeted release of active ingredients with the highest possible precision. One of the most remarkable examples in this context is probably the novel drug cannabidiol (CBD) [37], which is often applied after being dissolved in olive oil. CBD is a versatile drug, which is a potential treatment candidate for divers diseases [38] and its physiological effects range from anti-inflammatory properties [39,40,41], over anti-cancer activity [42,43,44] to protection against neurodegenerative diseases like Parkinson [45,46]. However, as pointed out in the literature, the quality of CBD may vary immensely depending on its source and the labelling of the extracts is not always sufficient [47] particularly if an average online vendor is chosen. Hence, without a reliable carrier system provided to and by pharmaceutical industry, it will remain difficult for CBD to unfold its full potential as a therapeutic substance, which outlines the necessity for further research on this topic.

In our study, we investigate the general ability of acrylamide-based microgels to act as carriers for CBD by scrutinizing its uptake and release using a two fold strategy. Firstly, we will try to utilize the hydrophobic interior of the microgel particles to generate partial solubility of CBD in the microgel suspension. Additionally, a solvent exchange route has been developed, where the loading of the microgels with CBD is performed in ethanol (EtOH). The main issue regarding the use of ethanol as a solvent for the microgel particles is their loss of thermosensitivity compared to microgels in water. Thus, it is important to ensure that the loading of microgels with CBD in ethanol is successful and no loss of CBD occurs. As a further improvement of the presented approach, the microgels loaded in ethanol are to be subjected to a solvent exchange to water, and once more, the loading with CBD and its release are investigated subsequently to the solvent exchange.

The study is employing different spectroscopic methods, in particular, UV/Vis spectroscopy to determine the amount of CBD inside the microgel network and transferred via temperature dependent release. Moreover, the influence of CBD loading into the microgel particles on their structure is investigated via atomic force microscopy (AFM). The carrier particles are tailored by the copolymerization of NIPAM and NIPMAM to exhibit a VPTT slightly above the body temperature of about 36 ${}^{\circ }C$ to 37 ${}^{\circ }C$, potentially allowing their use for hyperthermia treatments. As described earlier, CBD is a natural product and may contain different impurities depending on its source. Therefore, we additionally performed a thorough characterization of the CBD that we obtained, prior to the loading and release experiments (see Appendix A).

## 2. Results and Discussion

### 2.1. UV/Vis Spectroscopy of CBD

**Figure 2:**
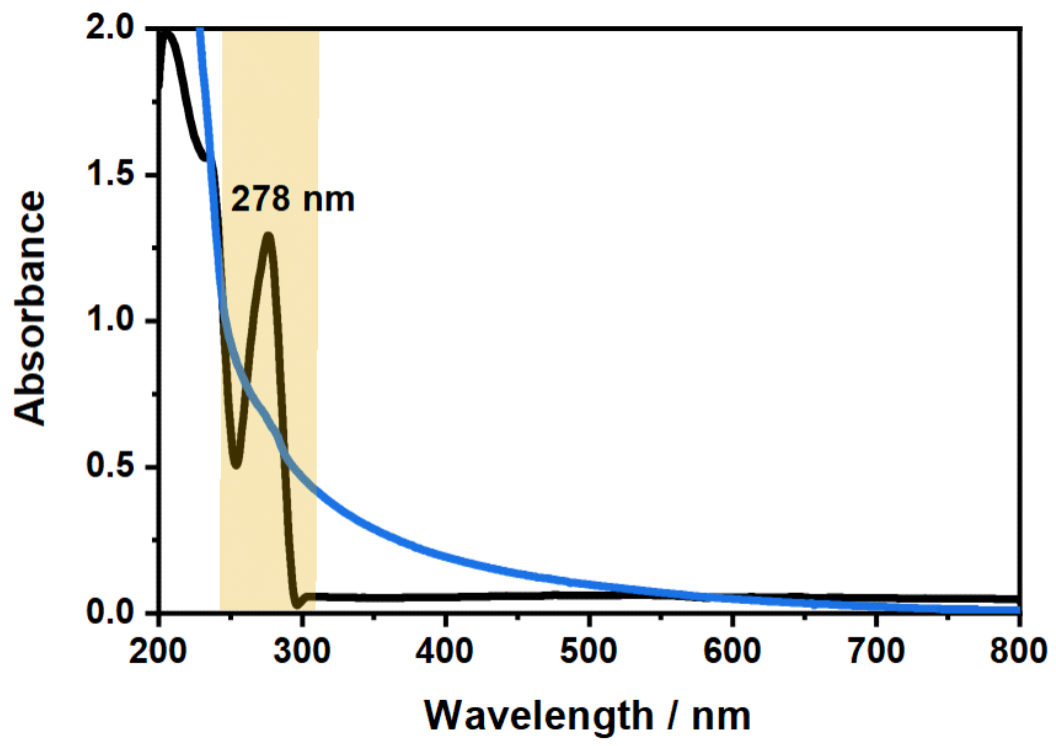
Comparison of the absorbance spectra of CBD (black) measured at a mass concentration of 0.1wt% in EtOH and the copolymer microgel (blue) measured at a mass concentration of 0.3wt% in water. The position of the exclusive absorbance band of CBD, which can be used to investigate the loading and release inside the microgel network, is indicated.

After verifying the purity of the used CBD sample (see Appendix A), we performed UV/Vis spectroscopy on pure CBD dissolved in ethanol (EtOH) to determine the most promising absorbance band of CBD for the investigation of its incorporation and release. Figure 2 shows the comparison of the UV/Vis absorbance spectra measured for the CBD and a typical copolymer microgel.

**Figure A1:**
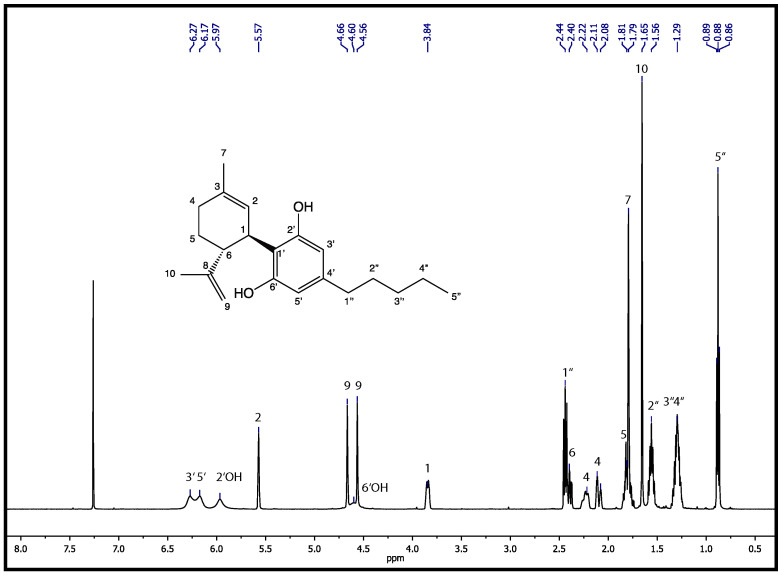
${}^{1}$H-NMR spectrum of the CBD sample, which was obtained and used for the loading and release experiments. The measurement was performed in ${\mathrm{CDCl}}_{3}$.

The main difference between the two absorbance spectra of CBD and the copolymer microgel is the characteristic absorbance band at 278 nm. In contrast to the wavelength dependent increase of the microgel absorbance, which is only caused by the light scattering from the particles and does not contain any molecular absorbance, a clear electron excitation band can be observed for CBD. Moreover, the observed absorption band in the spectrum can be assigned to the resorcinol substructure of CBD (see Figure A1 in Appendix A), which has also been observed in literature before.

**Figure A3:**
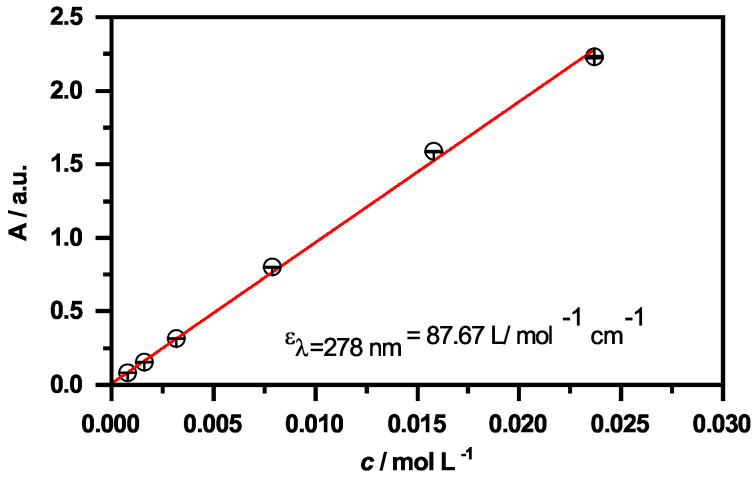
Absorbance at λ = 278 nm as function of the CBD concentration obtained from the UV/Vis measurements. The extinction coefficient is obtained from the slope of the linear fit (red line).

Due to the characteristic and very distinct absorption maximum of CBD at 278 nm, it will be possible to quantify the amount of CBD, that is released by the microgel particles using the same method. Therefore, we measured the absorbance spectra of CBD at various concentrations in EtOH and scrutinized the molecular extinction coefficient $\epsilon_{278}$, which exhibits a value of $87.67\frac{L}{\mathrm{mol}\cdot \mathrm{cm}}$. The respective calculation and figure can be found in the Appendix A in Figure A3. It is worth noting at this point, that the linear relation of the absorbance and concentration was not drastically deviating from the projection of the Lambert–Beer law, even though the absorbance exceeded a maximum value of 1, particularly at higher CBD concentrations. This indicates that the determination of the released amount of CBD could be determined over the whole concentration range used in the present study.

### 2.2. Initial Microgel Characterization

**Figure 3:**
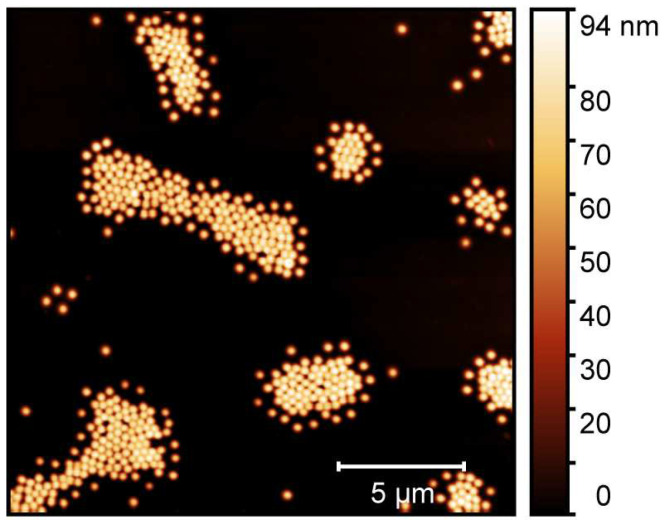
Atomic force microscopy image of adsorbed copolymer microgel particles (P(NIPAM-*co*-NIPMAM)) cast from water. The measurements have been performed in the completely dried state on a PEI-coated silicon surface.

The microgel particles obtained by a surfactant assisted precipitation polymerization were characterized in detail by means of atomic force microscopy (AFM), performed on adsorbed microgels in the completely dried state. The results of the AFM measurements of the particles are presented in Figure 3.

In the AFM image, we observe the microgel particles with a circular cross section adsorbed onto a silicon wafer surface. To ensure the successful sample preparation on the surface, it was coated with a thin layer of polyethyleneimine (PEI) prior to the deposition of the microgel particles [48]. Due to the favourable interactions of the PEI layer and the microgel particles, a strong flattening of the copolymer particle on the surface is observable [49,50]. Hence, the lateral extension of the particles appears to be much higher than the particle height, which is reflected in the scale shown next to the image. From a particle size analysis of at least 50 particles to ensure statistical significance, we obtained the average lateral radius of the copolymer microgels (100 nm), and their average height (100 nm) as well. Surely, the particle spreading on the surface is an important parameter to determine how the microgels interact with the surface during solvent annealing, and we will discuss this point in more detail at a later part in this study. At this point, it is most important to notice that we did not observe any sign of a second particle species, which would be indicative of non-successful copolymerization and of a secondary nucleation process. Instead, the sample of the copolymer microgel exhibits a narrow particle size distribution, which is typical for the employed surfactant assisted precipitation polymerization [15,51]. Hence, we suggest that the copolymerization of both monomers was successful and yielded the desired copolymer microgels. This can be confirmed by the investigation of the temperature-dependent swelling behaviour of the particles [15].

**Figure 4:**
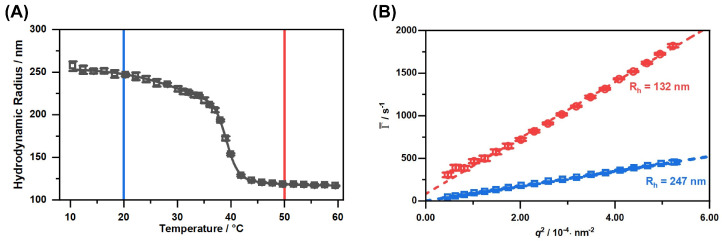
Hydrodynamic radius of the copolymer microgels obtained by temperature-dependent PCS measurements (**A**) and average relaxation rates $\bar{\Gamma }$ plotted against $q^{2}$ (**B**) measured with angle-dependent PCS at the temperatures 20 ${}^{\circ }C$ (blue) and 50 ${}^{\circ }C$ (red). The straight black line represents a sigmoidal fit through the swelling curve, employed to determine the VPTT of the copolymer microgel by using the point of inflection of the fit. Dotted coloured lines are linear fits used to determine the translational diffusion coefficient.

Subsequently, we performed temperature-dependent photon correlation spectroscopy (PCS) measurements to characterize the copolymer microgels in the dispersed state. Additionally, we investigated the particles with angle-dependent PCS measurements at two temperatures in the completely swollen (20 ${}^{\circ }C$) and fully collapsed (50 ${}^{\circ }C$) state. Figure 4 shows the swelling curve and the corresponding angle-dependent measurements of the copolymer microgel in water.

The swelling curve of the microgels (A) depicts the typical behaviour for most copolymer microgels of PNIPAM and PNIPMAM, which has been described by Keerl et al. [52]. The VPTT of the system at 39 ${}^{\circ }C$ was determined as the point of inflection of the sigmoidal fit of the swelling curve. This VPTT is very useful for the hyperthermia treatment of patients due to the proximity to the body temperature. Of course the microstructure of the copolymer microgel particles is not completely homogeneous in the region of the VPTT. Keerl et al. described a so-called “dirty-snowball” structure for the microgel particles in this temperature region, representing different network densities on a local scale [53]. Nevertheless, the angle-dependent PCS measurements in the fully collapsed and swollen state (B) yield a linear relation of the average relaxation rates and the square of the magnitude of the scattering vector. In both cases, the y-axis intercept is 0, so we conclude that the only relaxation process observed in the system is the diffusion of a single microgel particle species. Additionally, the calculated hydrodynamic radii of the angle-dependent measurements matches the values observed for the temperature-dependent measurements at a fixed angle of 45${}^{\circ }$. In combination with the AFM images and the circular cross-section of the particles in these images, we conclude that the particle synthesis yielded a single distribution of spherical copolymer microgel particles, with a VPTT exactly at the desired temperature, slightly above the body temperature.

**Figure 5:**
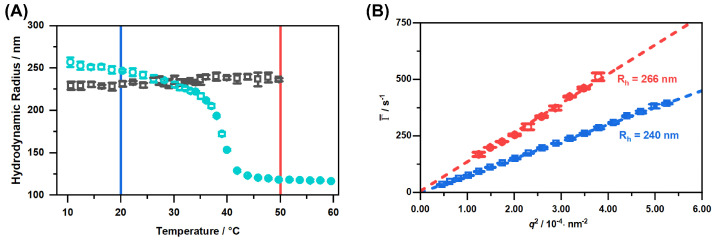
Hydrodynamic radius of the copolymer microgels obtained by temperature-dependent PCS measurements. The black squares represent the result of the measurements in EtOH and the blue circles the result from the measurements in water, given for comparison (**A**). Average relaxation rates $\bar{\Gamma }$ plotted against $q^{2}$ (**B**) measured with angle-dependent PCS in EtOH at the temperatures 20 ${}^{\circ }C$ (blue) and 50 ${}^{\circ }C$ (red). Dotted coloured lines are linear fits used to determine the translational diffusion coefficient.

We expected some difficulties in the loading and release process inside the microgel particles in water, because CBD is an extremely hydrophobic substance with almost zero solubility in water. Therefore, we prepared an alternative loading route, employing ethanol as a solvent for the microgel particles. To ensure complete solvent exchange, we performed numerous consecutive centrifugation, redispersion cycles in ethanol until no water was left in the system. The main problem we anticipate here is that pure ethanol is a good solvent for PNIPAM chains, regardless of the temperature at most concentrations [54]. Of course a mixture of water and ethanol would be even more problematic, because of the well-known cononsolvency effect, that has been extensively investigated by Richtering [55,56] and others [57]. Nevertheless, we investigated the temperature and diffusion behaviour of the microgel particles in EtOH by means of temperature and angle-dependent PCS measurements in the same way as the experiments have been performed for the same system in water. The result is shown in Figure 5.

It is clearly observable in the swelling curve (Figure 5A), that the microgel particles do not have any drastic volume phase transition on the scale of the particle radius in the ethanol mixture. In contrast to that, we observe a slight, continuous growth of the particle size. This difference becomes increasingly apparent, if we consider the swelling curve in water, which is shown for comparison. Unsurprisingly, we observe the same trend in the angle-dependent PCS measurements (right). From the slope of the linear regressions in this plot, we can confirm a slight increase in the particle size. Furthermore, we observe a slight deviation to higher values of the hydrodynamic radius compared to the temperature-dependent experiments.

**Figure 6:**
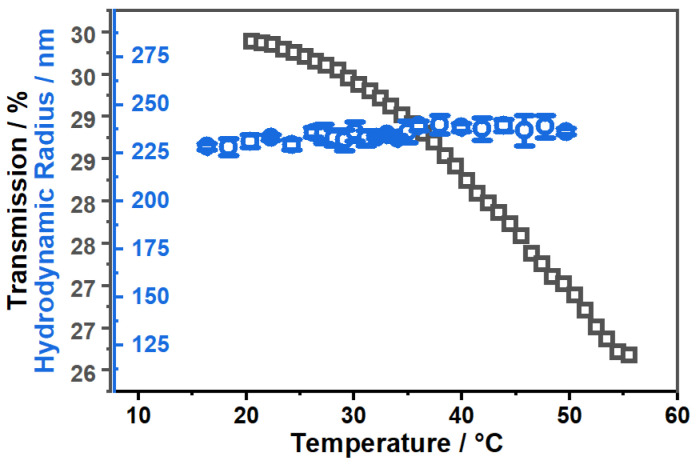
Comparison of the temperature-dependent development of the hydrodynamic radius (blue) measured by PCS at fixed scattering angle and the transmission (black) of the copolymer microgel suspension in EtOH measured by UV/Vis spectroscopy.

Subsequently, we performed some temperature-dependent UV/Vis measurements of the copolymer microgel particles in EtOH to investigate if the swelling that we observe in the measurements could be comparable with effects observed recently by von Klitzing et al. [58]. While observing the alcohol intoxication of microgel suspension with different techniques, they found a very interesting re-swelling behaviour, which was classified by them as ’non-typical UCST behaviour’. Of course, this has not been observed for microgels dispersed in pure ethanol so far, but on the other hand, the mentioned study focused rather on the variation of alcohols than on the investigation of copolymer microgels. Figure 6 shows a comparison of the results of the temperature-dependent turbidity measurements and the swelling curve of the microgel particles.

As a result of the measurements, we observe a constant decrease in transmittance, which usually occurs in correlation with swelling of the microgel particles upon temperature increase and has not been observed before in comparable systems dispersed in pure ethanol. It is important to note that the observed effect for the decrease in transmission is rather small (4%), but it is consistent throughout the measurement and was also observed in several repeated measurements.

As a summary of the previous section, it can be stated that the copolymer microgel synthesis was successful and we obtained particles exhibiting the desired VPTT and size. By anticipating potential issues with the CBD loading and release in water, we developed an alternative route which includes the dispersion of the copolymer particles in ethanol. As expected, PCS measurements show the non-existence of LCST behavior in EtOH. Instead, we observe a continuous swelling of the particles upon temperature increase. During the investigation of this size increase, we considered the possibility of a rather complex swelling behaviour, which has been discussed in literature recently for microgels in solvent mixtures. However, we have not been able to reveal the complete picture behind the mentioned effect yet, but it could be a hint that the temperature-dependent CBD uptake and release will potentially be possible with our alternative method, if we are not able to use the microgel system in water. Additionally, the microgels can be loaded in ethanol and subsequently transferred back to water, which allows temperature-triggered release.

### 2.3. Incorporation and Release of CBD into Thermoresponsive Microgels

**Figure 7:**
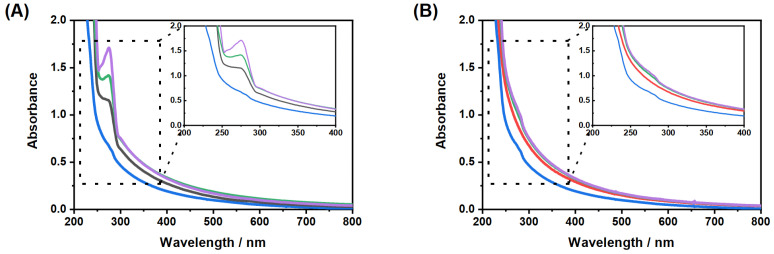
UV/Vis measurements for the investigation of the loading (20 ${}^{\circ }C$) and release (40 ${}^{\circ }C$) of CBD by the copolymer microgel particles. The measurements were performed after the incorporation (**A**) and release (**B**) of CBD in EtOH in a 0.3wt% microgel suspension. The initial concentrations of CBD were 0.5 mg/mL (black), 15 mg/mL (green) and 25 mg/mL (purple) (1 mg/mL and 5 mg/mL are not displayed for the sake of clarity of the presentation). The absorbance spectrum of a pure microgel suspension is given for comparison (blue).

**Figure A4:**
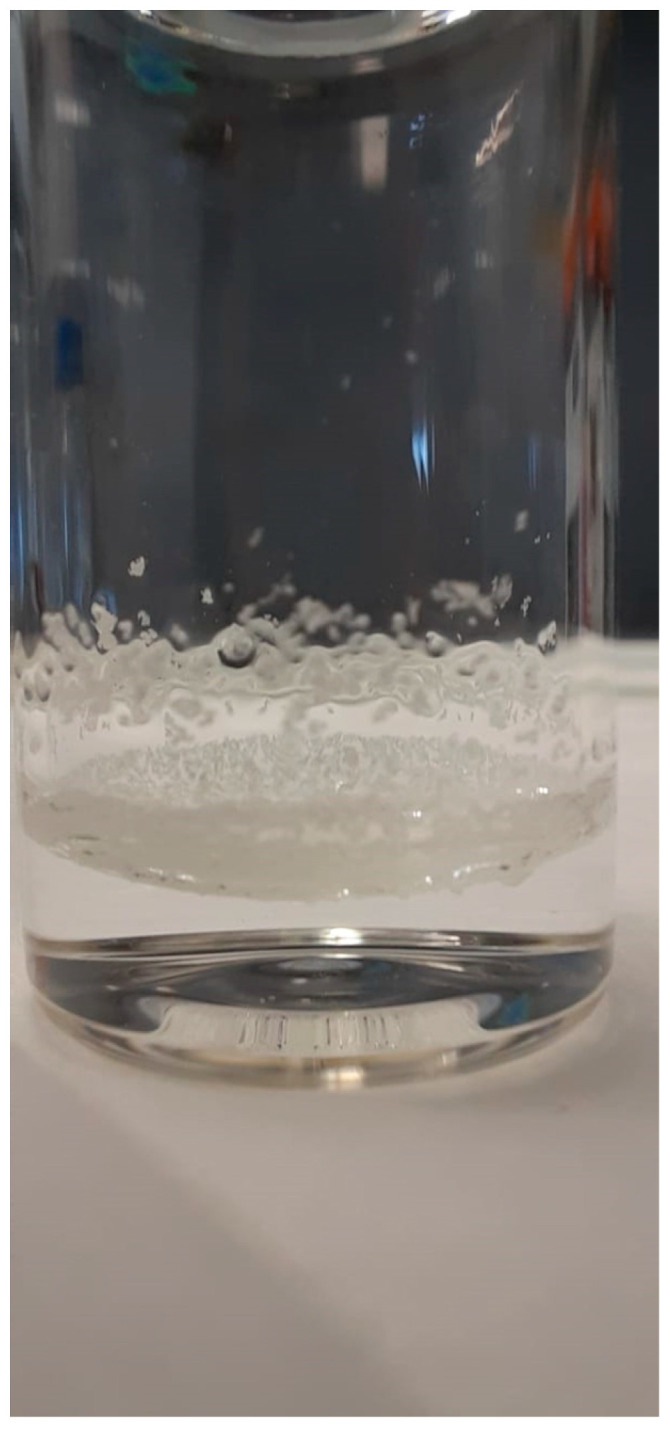
Photograph of CBD (20 mg/mL) dissolved in purified water. It can be clearly seen that the CBD does not dissolve in water. Rather, it sticks as a gel-like substance on the glass walls.

Firstly, we performed loading experiments with CBD in the aqueous microgel suspension by trying to dissolve the CBD inside the microgel particles in water. Right from the beginning, it became obvious that the enormous hydrophobicity of CBD does not allow the usage of this approach. Even after centrifugation, the substance floats on top of the water surface, without dissolving at all (see photography in Figure A4). Due to this issue, we focused on our alternative incorporation route for the CBD employing ethanol as a solvent. The general procedure was to dissolve both substances in EtOH, then centrifuge the microgel particles. After decantation and redispersion, the incorporation of CBD was measured with UV/Vis spectroscopy (Figure 7A). The loading, centrifugation as well as the measurement were performed at a temperature of 20 ${}^{\circ }C$.

Figure 7A shows a significant increase in the optical density of the band which is characteristic for CBD, with increasing CBD concentration compared to the pure microgel. Due to the fact that the microgel particles also contribute to the absorbance of the pure CBD in the loaded systems, the determination of the absorbed concentration of CBD is difficult. For this purpose, we subtracted the spectrum of the pure microgel particles from the loaded systems and calculated the approximate amount of CBD absorbed at the different concentrations by the microgel using the previously determined extinction coefficient. The results are summarized in Table 1.

**Figure A5:**
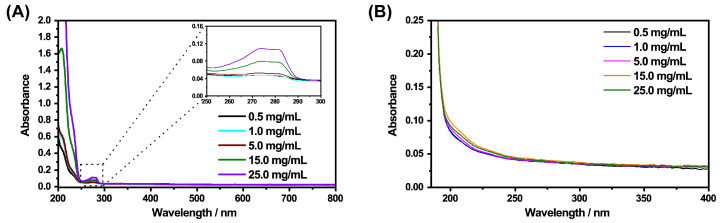
UV/Vis measurements of the supernatants of the loaded microgels at 20 ${}^{\circ }C$ to study the bleeding of CBD after centrifugation and the re-addition of ethanol (**A**). Supernatant of the loaded microgels (20 ${}^{\circ }C$) after centrifugation and the addition of water (**B**).

It is interesting that at low initial CBD concentrations of 0.5 mg/mL and 1 mg/mL, the CBD is completely incorporated into the microgel particles. By increasing the initial concentration, the incorporation of the CBD decreases compared to the provided amount. From an initial concentration of 25 mg/mL, only about 12% (2.9 mg/mL) are adsorbed. It seems that the concentration of the microgel (0.3wt%) is insufficient to ingest more CBD. Usually, it would be a simple strategy to increase the mass concentration of the microgel, which can easily be done because the mass concentration which supposedly results in the formation of colloidal crystals is far above the concentration used here. However, we did not increase the microgel concentration to focus on a set of comparable experiments in the present study. Nevertheless, a clear trend for the increase in CBD incorporation with increasing initial CBD concentration can be observed, even if, as mentioned above, not as much is incorporated at higher initial concentrations as would initially be expected, but still more than at low concentrations. To exclude the release of CBD in ethanol at 20 ${}^{\circ }C$, we also examined the supernatant at this temperature after centrifugation. The results are shown in Figure A5A. It can be seen that the small amounts of CBD are released even at a temperature of 20 ${}^{\circ }C$. However, compared to the release performed at 40 ${}^{\circ }C$, the amount is very small and can be attributed to the “bleeding” of CBD out of the microgel network when ethanol is added again before centrifugation.

**Figure 8:**
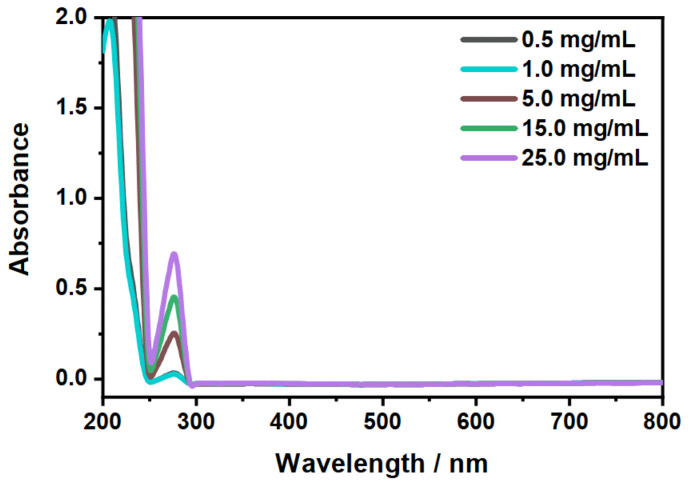
Measurement of the temperature-dependent release (40 ${}^{\circ }C$) of CBD in ethanol. The legend displays the initial CBD concentration. The extinction coefficient at λ=278nm, determined during the initial CBD characterization, was used to calculate the efficiency of the release.

To release the CBD in EtOH, the solution was heated to 40 ${}^{\circ }C$ and the copolymer microgel particles were removed via centrifugation. Another set of UV/Vis measurements was performed after subsequent exchange of the EtOH to measure the remaining amount of CBD inside the network (Figure 7B). In addition, the removed supernatant after the release was also investigated for the released amount of CBD by means of UV/Vis measurements (Figure 8). The obtained results are also listed in Table 1.

**Figure 9:**
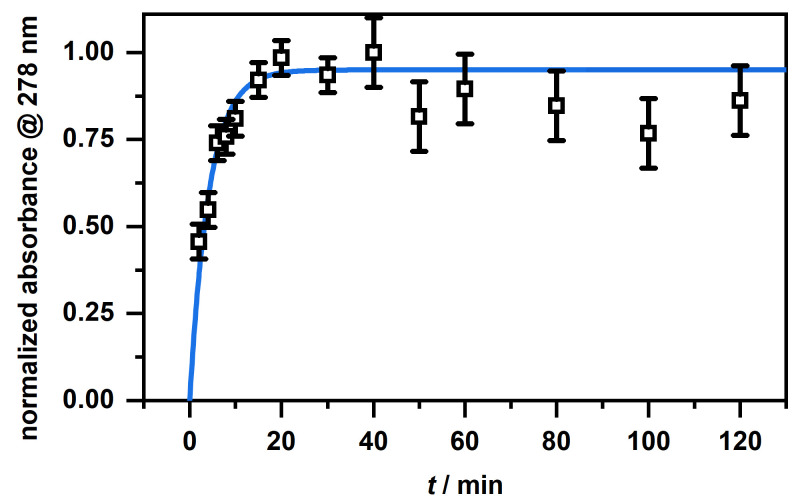
Time-dependent release of CBD in EtOH at 40 ${}^{\circ }C$ as a plot of the normalized absorbance at 278 nm, obtained from the UV/Vis measurements, against time.

At first glance, the microgels in Figure 7 B show a slightly higher absorbance after the release compared to the unloaded microgels (blue). This is due to residual CBD, which is not released from the particles. This becomes clear when looking at the spectra of the supernatant after the release (Figure 8) and at the corresponding calculated concentrations from Table 1. First of all, it can be seen that, as the initial concentration increases, the absorbance for the released CBD also increases. This also corresponds to the previously discussed loading of CBD. If the determined concentrations are now considered, it can be seen that the CBD is not completely released. Some CBD remains in the polymer network, particularly at low initial concentrations of 0.5 mg/mL and 1 mg/mL. The release increases when the initial concentration is increased, and in all three cases, is around 80% of the previously ingested CBD. This result is very surprising considering that the copolymer microgel particles do not exhibit LCST behavior when dispersed in EtOH. As discussed in the previous section, the incorporation of CBD potentially influences the solvent–polymer interactions in our polymer system and therefore enables the carrier function of the copolymer microgel particles just under the given circumstances. In addition, it can be assumed that the solubility of CBD is significantly increased at high temperatures, resulting in a significant contribution to the release, since, as already shown in the additional measurements at 20 ${}^{\circ }C$ (Figure A5A), small amounts are released from the network via the re-addition of ethanol. For this purpose, we additionally investigated the time-dependent release of CBD at 40 ${}^{\circ }C$ in ethanol. The results are shown in Figure 9. The release as a function of time shows that, after a few minutes, a clear increase in the released CBD can be detected in solution, with the maximum value being reached after approx. 20 min. Thus, the amount of CBD released can actually be attributed to the temperature effect. This is of course very interesting, and consequently, we investigated the interaction and particularly the structure of the copolymer microgels with adsorbed CBD molecules in more detail.

**Figure 10:**
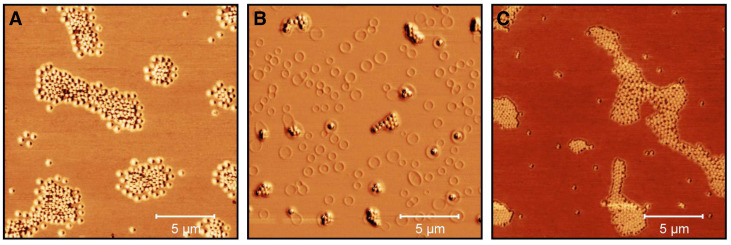
AFM phase images (20 × 20 μm) of microgel particles in water (**A**), microgel particles in ethanol/CBD solution ($c_{CBD}$ = 25 mg/mL) (**B**) and after the release of CBD in ethanol at 40 ${}^{\circ }C$ (with removed supernatant and redispersion in ethanol) (**C**). The images were recorded at room temperature in the dried state on PEI coated silicon wafers.

To do so, we performed AFM examinations of the microgels deposited from ethanol solution and after the incorporation of the CBD in ethanol to investigate possible changes in the microgel structure. The phase images are particularly interesting here and therefore these are summarized in Figure 10 for the microgels cast from water (A), an ethanol/CBD solution (B) and ethanol (after the release) (C).

**Figure 11:**
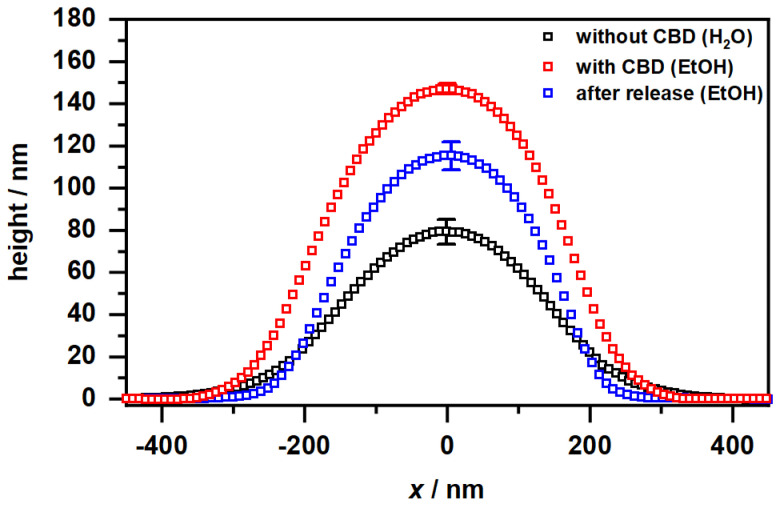
Height profiles of the copolymer microgel particles cast from water (black), after CBD incorporation deposited from EtOH (red) and after CBD release and again cast from EtOH (blue), obtained by AFM measurements in the completely dry state after the deposition of the copolymer microgel particles on a PEI coated silicon surface. All height profiles were generated by averaging the individual height images of at least 50 particles. The error bar indicates the statistical uncertainty of the height.

**Figure A6:**
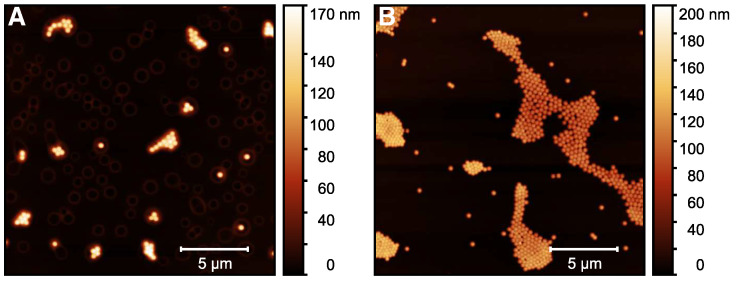
AFM height images (20 × 20 μm) for the microgel particles in ethanol/CBD solution (**A**) and after the release of CBD in ethanol solution (**B**). The measurements were performed in the dried state at room temperature. The images were used to extract height profiles of the microgel particles using the software GWYDDION.

**Figure A7:**
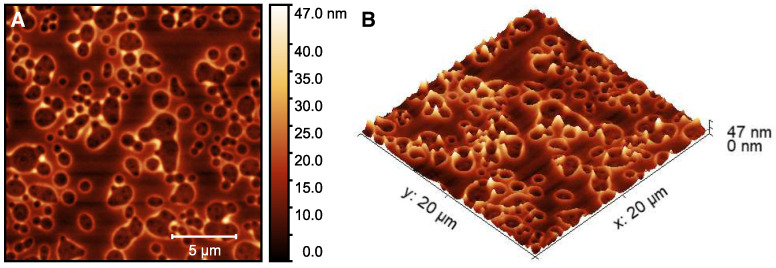
AFM height image (20 × 20 μm) of an ethanol/CBD solution (**A**) and the corresponding 3D image (**B**). The measurements were performed in the dried state at room temperature.

Figure 10A shows the expected appearance for copolymer microgel particles fully dried on a silicon substrate. These are particles with a spherical cross section exhibiting a slight “fuzziness", which can be observed as a typical phase difference between the particle interior and the outer area. If the microgels are now dispersed in an ethanol/CBD solution and dried, interesting observations can be made (Figure 10B). Annular structures of different diameters can be observed at different positions on the wafer, regardless of the presence of copolymer microgel particles. Furthermore, the particles appear much more rigid, which can be deduced from the increased phase difference and the disappearance of the previously observed “corona". If additionally the height profiles of the particles extracted from the corresponding height images (see Section 2.2 and Figure A6) are considered (Figure 11), it becomes clear that the previously rather “soft” particles, which spread well on the substrate surface, now become significantly more rigid (height approx. 80 nm vs. 145 nm) and collapse less in the adsorbed state. This suggests that CBD is stored in the particles and thus confirms the results from the UV/Vis measurements. The ring-shaped structures described above can most-likely be assigned to the interaction of the CBD with the wafer (PEI) surface. We suppose that these objects represent unbound CBD molecules forming some sort of organized structures due to the solvent annealing process of ethanol. This observation can also be made in Figure A7, where a pure CBD/EtOH solution on a wafer was investigated by AFM measurements.

Compared to the previously discussed phase images, in the phase image of the copolymer microgel particles after the release of CBD into ethanol (Figure 10 C), a significant difference to the microgels in water can be observed, since they still do not show any “fuzziness”. Hence, it seems clear that this phenomenon can be assigned to different interactions between the substrate and the copolymer microgel particles deposited from water and ethanol. This nicely agrees with the results from the PCS measurements, as we also found different hydrodynamic radii in the fully swollen state in these measurements (Figure 4B and Figure 5B). However, it is worth noting that maybe the remaining CBD, which has not been released by the microgel as shown in the UV/Vis measurements plays a role in this context. Furthermore, the particles also have a different spreading behavior, if they are investigated after the CBD release or measured when deposited from water. If the height profile is observed, a clear decrease in the height of the particles to approx. 120 nm can be observed, compared to the fully CBD loaded copolymer microgel particles. Nonetheless, the particles are more rigid than the ones deposited from pure water, which initially agrees with the PCS measurements.

**Figure 12:**
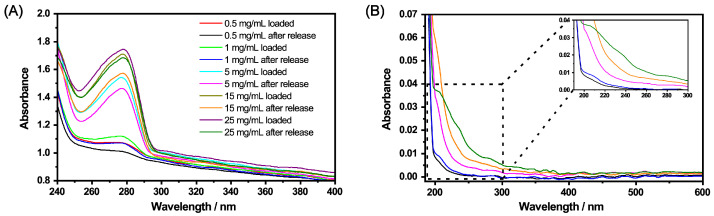
UV/Vis measurements of the copolymer microgels loaded with CBD and after the release of CBD (**A**). The loading was conducted in ethanol at 20 ${}^{\circ }C$, then the solvent was completely removed and replaced by water. CBD in the supernatant after the release (40 ${}^{\circ }C$) in water (**B**). The concentrations in (**B**) correspond to the colours after the release in (**A**).

The results obtained so far show that the microgels have been successfully loaded with CBD at 20 ${}^{\circ }C$ and that CBD can also be released by changing the temperature to 40 ${}^{\circ }C$. However, ethanol as a solvent is rather poorly suited for the use in drug carrier systems in the human body. Therefore, we were dedicated to further improve our approach for loading the microgels in ethanol by adding another solvent exchange step subsequent to the ethanol assisted loading. Consequently, we removed the loaded microgels from the solution and redispersed them in water repeatedly, until no ethanol was left. The UV/Vis measurements of the supernatant at 20 ${}^{\circ }C$ in water show that there is no “bleeding” of CBD from the microgel network compared to the microgels in ethanol (see Figure A5B in the Appendix A). The results for loaded microgels (20 ${}^{\circ }C$), microgels after the release of CBD (40 ${}^{\circ }C$) and the released CBD in the supernatant in pure water are shown in Figure 12.

Figure 12A shows the characteristic band of CBD for loaded microgels and microgels after the release of CBD. First of all, the trends already described for the loading in ethanol are preserved and the concentration dependence of the initial CBD concentration is retained. This indicates a successful transfer of the loaded microgels into water without losing a significant amount of CBD throughout the process steps. It is also noticeable that after the release of CBD, the absorbance of CBD inside the microgel particles decreases for each concentration of CBD compared to the loaded state. However, of course some of the CBD still remains inside the particles. The decrease in the CBD absorption inside the microgel particles suggests that a certain amount of CBD was released from the network into water. This observation can also be confirmed when the supernatant of the release solution is investigated (Figure 12B). Unfortunately, in this case, it is not possible to precisely scrutinize the amount released, due to the use of the Ulbricht sphere for the measurements. However, nonetheless, we could show that some of the CBD is released in water.

**Figure 13:**
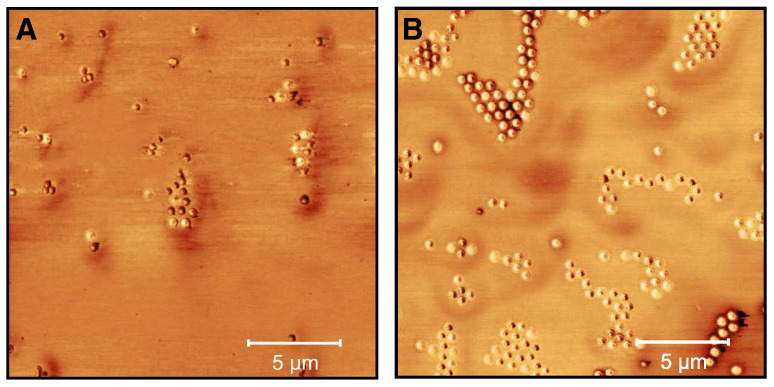
AFM phase images (20 × 20 μm) of microgel particles loaded with CBD ($c_{CBD}$ = 25 mg/mL) in water (**A**) and after the release of CBD in water (with removed supernatant and redispersion in water) (**B**). The images were recorded at room temperature in the dried state on PEI-coated silicon wafers.

In order to further characterize the microgels loaded with CBD in water and after the release, AFM measurements were performed. Figure 13A shows the phase image of the loaded microgel particles. It is noticeable that the incorporation of CBD into the microgels is not homogeneous, since the phase difference between the particles is altered. Most of the microgels show a strong phase difference. With some particles, however, it can be seen that the CBD is ingested into the center of the polymer network and a slight core-corona structure is present. After the release of CBD, a clear change in the particle structure is recognizable (Figure 13B). Most of the particles are no longer compact and show a core-corona like structure, as is known from unloaded microgels [49,50]. Furthermore, it can be seen that a small phase difference appears in the center of the particles, which indicates that a certain amount of CBD remains in the microgel network and agrees well with the UV/Vis measurements. In addition, microgels without a phase difference are present, which probably indicates a varying release of CBD throughout the sample.

**Figure 14:**
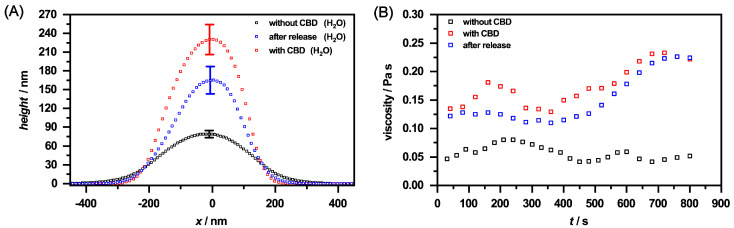
(**A**) Height profiles of the copolymer microgel particles in water (black), after loading with CBD (red) and after CBD release (blue), obtained by AFM measurements in the completely dry state after deposition of the copolymer microgel particles on a silicon surface. All height profiles were generated by averaging the individual height images of at least 30 particles from Figure A8. The error bar indicates the statistical uncertainty of the height. (**B**) Rheology measurements of the corresponding microgel particles at a constant shear rate of 0.5 s${}^{-1}$ and a duration of 800 s. The change in the viscosity of the different microgel solutions is shown as a function of time. The measurements were performed at a temperature of 20 ${}^{\circ }C$.

**Figure A8:**
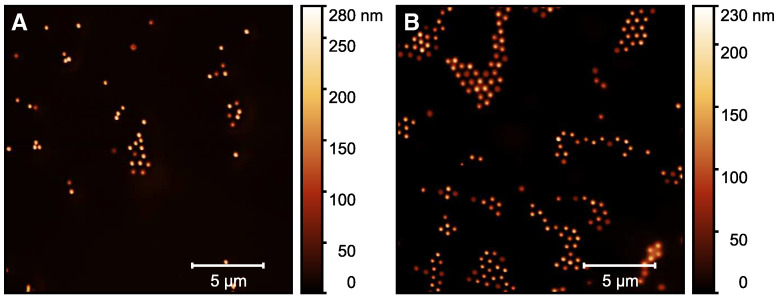
AFM height images (20 × 20 μm) for the microgel particles loaded with CBD (**A**) and after the release of CBD in water (**B**). The measurements were performed in the dried state at room temperature. The images were used to extract height profiles of the microgel particles using the software GWYDDION.

These observations are additionally supported by the height profiles for the corresponding microgels (Figure 14A) obtained from Figure A8. At first glance, the microgels in the loaded state exhibit the same lateral size as the unloaded microgels, but it has to be taken into account that these are significantly more rigid and have a height of approx. 240 nm compared to the unloaded system with approx. 80 nm. After the release of CBD, the particles still show a certain rigidity, with the difference being that the height of the particles decreases significantly, which is in line with the release of CBD, observed in the UV/Vis measurements. Moreover, a similar trend can be observed in the height profiles as for the microgels dispersed in ethanol. As already shown by the phase images, it can also be seen here that a certain amount of CBD still remains in the microgel network. It is also noticeable that the loaded particles and the microgels after the release of CBD demonstrate a slightly higher rigidity compared to the microgel particles in ethanol. We suspect that this effect is caused by the solvent exchange to water and its interaction with the very hydrophobic CBD, which causes the polymer network to become stiffer. The differences between the microgels in different loading states of the particles can also be clarified by rheological measurements. Therefore, we investigated the viscosity of a defined amount of the microgels as a function of time at a constant shear rate (B). It is noticeable that the viscosity for the unloaded microgel remains almost constant. The loaded system already shows a significantly higher viscosity at the beginning, indicating a change in the morphology of the polymer network due to the ingested CBD. After approx. 500 s, there is an increase in viscosity, which indicates a higher structural order of the particles due to the shearing of the sample. The viscosity of the microgels after the release of CBD, on the other hand, is slightly lower, but follows the same trend by reaching a plateau, which is caused by the residual CBD not released from the microgels.

In any case, from the AFM measurements and the additional rheological investigations, it can be concluded that the interactions of the CBD and the polymer network have a very important influence on the particle structure, which is even detectable, after the release of large amounts of the CBD from the network. Thus, it seems reasonable to us that the temperature-induced release of the CBD in ethanol and water is not purely coincidental for our microgel system, but seems to be the result of a complex interaction pattern of all materials and solvents involved in the process.

## 3. Materials and Methods

### 3.1. Materials

*N*-isopropylacrylamide (NIPAM, TCI Germany GmbH, Eschborn, Germany, 97%) and *N*-isopropylmethacrylamide (NIPMAM, Sigma Aldrich, St. Louis, MO, USA, 97%) were recrystallized from *n*-hexane (VWR International, Darmstadt, Germany, p.a.). *N*,*N*′-methylenebisacrylamide (BIS, 99%), sodium dodecyl sulfate (SDS, 99%) and ammonium peroxodisulfate (APS, 99%) were purchased from Sigma-Aldrich (Munich, Germany) and used without further purification. Cannabidiol (CBD) was obtained as a sample from Cannovum AG (Berlin, Germany) and used as obtained.

### 3.2. Microgel Synthesis

All microgels were synthesized via a surfactant-assisted precipitation polymerization, first established by Pelton and Chibante [59]. Briefly, the monomers NIPAM (2.88 mmol) and NIPMAM (2.88 mmol) were dissolved in purified water (74 mL) alongside the cross-linker BIS (5 mol%) and the solution was vigorously stirred and degassed using a continuous nitrogen flow for one hour at 80 ${}^{\circ }C$. Briefly, prior to the initiation of the polymerization, SDS (0.05 mmol) was added. After a few minutes, the polymerization was started by adding 1 mL of an APS solution (0.20 mmol/mL, 3.5 mol%). Afterwards, the solution was further stirred at 80 ${}^{\circ }C$ for 4 h, cooled down to room temperature and stirred overnight. The purification of the microgels was carried out by five consecutive centrifugation, decantation and redispersion cycles with purified water (20,000 rpm, 45 min).

### 3.3. Loading and Release of CBD

The incorporation of CBD in EtOH at 20 ${}^{\circ }C$ was investigated using an Agilent 8453 UV/Vis spectrometer (Agilent Technologies Germany, Rattingen, Germany) equipped with a diode-array detector. The sample holder was kept at 20 ${}^{\circ }C$ by using a water thermostat (Julabo F25, Julabo GmbH, Seelbach, Germany). For the sample preparation EtOH solutions with a constant microgel amount (0.3wt%) and different CBD concentrations (0.5 mg/mL–25 mg /mL) with a total volume of 1 mL were centrifuged for 15 min (15,000 rpm, 20 ${}^{\circ }C$) using a Mikro 200 R centrifuge (Andreas Hettich GmbH & Co. KG, Tuttlingen, Germany). The supernatant was completely removed and replaced by the same amount of EtOH. In addition, a microgel sample without CBD was prepared using the same procedure. The solutions were transferred into Hellma cuvettes (Hellma GmbH, Mülheim, Germany, 1 mm) and equilibrated for 20 min. The absorbance was measured from 200 nm–800 nm. In addition to the measurements of the pure microgel in EtOH and the microgels loaded with CBD, pure CBD in EtOH was also measured at the same concentrations and temperature for the determination of the extinction coefficient (see Figure A3).

The same sample preparation was used to study the release of CBD in EtOH. The prepared samples with a total volume of 1 mL were centrifuged for 15 min (15,000 rpm). The supernatant was completely removed and replaced by the same amount of EtOH and centrifuged again for 15 min (15,000 rpm) at 40 ${}^{\circ }C$. The supernatants were transferred in a Hellma cuvette (Hellma GmbH, Mülheim, Germany, 1 mm). The absorption was measured from 200 nm–800 nm. In addition to the supernatant measurements, the microgel after the release of CBD was redispersed in 1 mL EtOH and measured the same way to check whether there are any residues of CBD in the microgels.

In addition, the release of CBD in ethanol at 40 ${}^{\circ }C$ was investigated in a time-dependent manner. For this purpose, the loaded microgel (2 mL) was placed in a dialysis tube (Spectra/Por Biotech CE Tubing 1000 kDa, Repligen, USA), which was immersed in a vessel containing ethanol (150 mL, 40 ${}^{\circ }C$) and 14 samples, 1 mL each, were taken from the dialysis medium under stirring in a time range of 0–2 h and examined by UV/Vis spectroscopy (see above). After each collection, 1 mL of EtOH was added to the dialysis medium.

To investigate the microgels loaded with CBD in an aqueous solution and after the release of CBD, the microgels were loaded with the appropriate CBD concentrations in EtOH as described above. Afterwards, the solvent was completely removed and the microgels loaded with CBD were redispersed in the same amount of water and transferred to Hellma cuvettes (Hellma GmbH, Mülheim, Germany, 10 mm). The cuvettes were placed in an Ulbricht sphere (Shimadzu Germany, Duisburg, Germany). The measurements were performed at room temperature using a UV/Vis spectrometer (UV-2450, Shimadzu Germany GmbH, Duisburg, Germany). The released amount of CBD in aqueous solution in the supernatant was investigated similarly to the release in EtOH.

### 3.4. NMR Spectroscopy

${}^{1}$H-NMR spectroscopy was performed using a FT-NMR spectrometer (Avance III 500, Bruker Corporation, Karlsruhe, Germany). The pure CBD (5 mg) was dissolved in CDCl${}_{3}$. The measurements were done at 500 MHz and 1024 scans. The obtained spectrum was analyzed by the software MestReNova. The assignment of the significant signals is summarized in Table A1.

### 3.5. Mass Spectrometry

Nano-ESI mass spectra were recorded using an Esquire 3000 ion trap mass spectrometer (Bruker Daltonik GmbH, Bremen, Germany) equipped with a nano-ESI source. The sample was dissolved in EtOH and introduced by static nano-ESI using in-house pulled glass emitters. Nitrogen served both as nebulizer gas and dry gas. Nitrogen was generated by a Bruker nitrogen generator NGM 11. Helium served as cooling gas for the ion trap and collision gas for MSn experiments. The mass axis was externally calibrated with ESI-L Tuning Mix (Agilent Technologies, Santa Clara, CA, USA) as calibration standard. MestReNova was used for processing the spectra.

### 3.6. Photon Correlation Spectroscopy (PCS)

For the determination of the particle size of the microgels, PCS measurements on highly diluted samples in ethanol or purified water were performed. Angle-dependent PCS measurements were done using a 3D-LS Spectrometer Pro (LS Instruments AG, Fribourg, Switzerland) equipped with a HeNe LASER (JDSU 1145P, Thorlabs Inc., Newton, NJ, USA) from 40${}^{\circ }$–100${}^{\circ }$ in steps of 5${}^{\circ }$ at 20 ${}^{\circ }C$ and 50 ${}^{\circ }C$. The primary beam was polarized. However, no analyzer was installed in front of the detector. Hence, a zero intercept in the plots of $\bar{\Gamma }$ vs. $q^{2}$ indicates the absence of rotational or deformation modes. The temperature was adjusted using a temperature controlled index matching bath. For each measurement, the sample was allowed to equilibrate for 25 min.

To study the temperature-dependent phase behaviour and to obtain the volume phase transition temperature (VPTT) of the microgels, PCS measurements at a fixed angle of 45${}^{\circ }$ were performed using a HeNe LASER (632.8 nm, Thorlabs, Newton, NJ, USA) with a 6010 multiple-τ digital auto-correlator (ALV, Langen, Germany) and a SO-SIPD single photon detector (ALV, Langen, Germany). The temperature was adjusted using a thermostated index matching bath. The measurements were performed in a temperature range from 10 ${}^{\circ }C$–50 ${}^{\circ }C$. At each temperature, the sample was allowed to equilibrate for 25 min.

The obtained auto-correlation functions were analysed using the method of cumulants [60] to obtain the mean relaxation rate $\bar{\Gamma }$. Via $\bar{\Gamma }=D^{T}\;\cdot \;q^{2}$, the translational diffusion coefficient $D^{T}$ can be calculated with *q* as magnitude of the scattering vector:

(1) $$q=\left|\vec{q}\right|=\frac{4\pi n}{\lambda }\;\cdot \;\sin\frac{\theta }{2}$$

with λ being the wavelength of the scattered light, *n* the refractive index of the solvent and θ the scattering angle. Using the Stokes–Einstein Equation (2) with the temperature *T*, the Bolzmann constant $k_{B}$ and the solvent viscosity η, a calculation of the hydrodynamic radius $R_{h}$ of the microgel particles is possible:

(2) $$D^{T}=\frac{k_{B}T}{6\pi \eta R_{h}}$$

### 3.7. Atomic Force Microscopy

Atomic force microscopy was used to investigate the microgel particles for structural changes depending on the used solvent (water or ethanol) and due to the incorporation of CBD into the microgel network. All measurements were performed with a DI Nanoscope IIIa (Digital Instruments, now Bruker, Karlsruhe, Germany) mounted on a Zeiss Axiovert 135 inverted microscope (Carl Zeiss Microscopy GmbH, Jena, Germany) in semi-contact mode using Budget Sensors (Innovative Solution Bulgaria Ltd., Sofia, Bulgaria) Al-Reflex Tap300Al-G cantilevers with a tip radius of <10 nm, a resonance frequency of about 300 kHz and a spring constant of 40 N/m. For the sample preparation, a silicon wafer (Siegert Wafer GmbH, Aachen, Germany) was cleaned with ethanol (HPLC grade) and subsequently in a plasma cleaner (Zepto, Diener Electronics GmbH, Ebhausen, Germany). Afterwards, the cleaned wafer was spin-coated with 0.1 mL of a PEI solution (0.25wt%), and then with a highly diluted microgel suspension (in water, ethanol or CBD/ethanol solution). The measurements were performed in the dried state at room temperature. The resulting images were analyzed with GWYDDION [61].

### 3.8. UV/Vis Measurements

Turbidity measurements on microgels in EtOH were performed using an Agilent 8453 UV/Vis spectrometer (Agilent Technologies Germany, Rattingen, Germany) equipped with a diode-array detector in the range from 20 ${}^{\circ }C$ to 55 ${}^{\circ }C$ with a heating rate of 2.5 ${}^{\circ }C$/h. The temperature of the sample holder was controlled by a thermostat (Julabo F25, Julabo GmbH, Germany). For the measurement, the microgel solution (0.1wt% in ethanol) was transferred into a Hellma cuvette (Hellma GmbH, Mülheim, Germany, 10 mm) equipped with a stir bar. The temperature-dependent measurement was recorded at a wavelength of 400 nm in a measurement interval of 20 s.

### 3.9. Rheological Measurements

To study the time-dependent change in viscosity of pure microgels, microgels loaded with CBD, and microgels after the release of CBD rheological measurements were performed using a Physica MCR 101 rheometer (Anton Paar Germany GmbH, Ostfildern-Scharnhausen, Germany) equipped with a cone plate shear cell. For the measurements 15 μL of the respective microgel solution (in water) was used. The viscosity was measured for 800 s at a constant shear rate of 0.5 s${}^{-1}$ at room temperature.

## 4. Conclusions

In this work, we used copolymer microgels for loading and release experiments of the hydrophobic drug CBD. Since loading of the microgels with CBD in water was not possible due to the hydrophobicity of the drug, we successfully developed an alternative incorporation route by redispersing the copolymer microgel particles in ethanol at 20 ${}^{\circ }C$. As expected, the microgel particles did not exhibit LCST behavior in ethanol. However, at higher initial CBD concentrations, the percentage of incorporated CBD obviously reaches a threshold value for the given microgel concentrations. Nonetheless, this issue can be addressed simply by using higher concentrations of microgels for the uptake. Furthermore, we also observed a temperature-dependent release of the CBD by the microgel particles at 40 ${}^{\circ }C$ in ethanol. Moreover, a re-transfer of loaded microgels into water, which surely is a much better solvent for the application of microgels as drug carrier matrices, was not only possible, but additionally allowed a temperature-dependent release of CBD into water by utilizing the microgels LCST. Finally, we were able to show via AFM measurements and viscosity measurements, that the CBD exhibits strong interactions with the microgel network and the subsequent release of CBD from the network leaves some of the CBD inside the network, resulting in different particle morphology.

Nevertheless, copolymer microgel systems have proven to be an extremely interesting carrier system for CBD and showed outstanding uptake when they were used in an appropriate solvent. In the end, it might be beneficial that CBD as a rather new drug does not have a perfectly developed carrier system so far, which opens up an interesting development opportunity for microgel carriers.

## Figures and Tables

**Table 1:** Initial CBD concentration used for the incorporation of CBD into the microgel network at 20 ${}^{\circ }C$ and a summary of the calculated concentrations of the ingested, released and remaining CBD concentration at 40 ${}^{\circ }C$ in EtOH in the microgel particles obtained from the calculation using the UV/Vis spectra.

Initial/mg mL${}^{-1}$	Uptake/mg mL${}^{-1}$	Release/mg mL${}^{-1}$	Remaining in Microgel/mg mL${}^{-1}$
0.5	0.50	0.11	0.44
1	0.99	0.09	0.90
5	1.00	0.80	0.20
15	1.90	1.50	0.40
25	2.90	2.30	0.60

**Figure A2:**
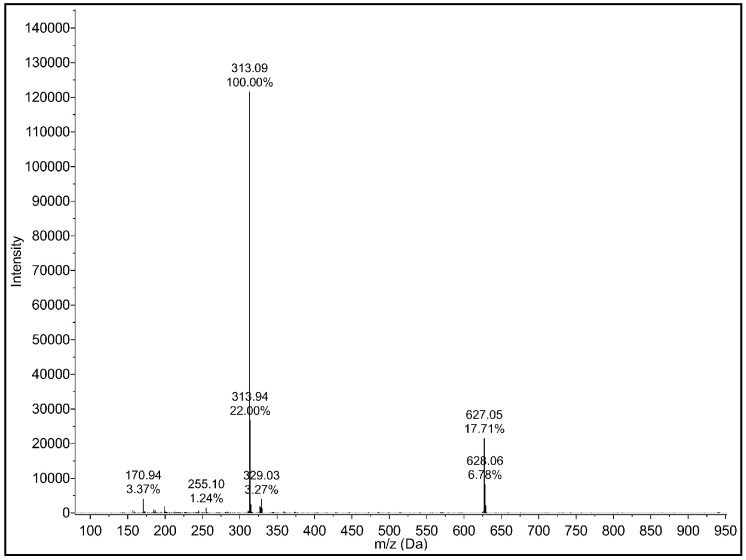
Mass spectrum of cannabidiol (CBD) recorded by ESI-MS in negative mode. The spectrum shows the molecular signal of CBD at *m/z* = 313.09 (exact mass of CBD = 314.46 g/mol).

## Data Availability

The data presented in this study are available on request from the corresponding author.

## Sample Availability

Samples of CBD and microgel are available on demand from the authors.

## Appendix A. Characterization of the CBD

As CBD is a natural product, it cannot be ruled out that even the most extensively purified material, as used in this study, contains small amounts of impurities. To verify the quality of the used CBD batch, which will be used for the loading and release experiments and the characterization of the mixed samples, ${}^{1}$H nuclear magnetic resonance spectroscopy (NMR spectroscopy) and electrospray ionization (ESI) mass spectrometry were performed to identify the potential impurities present in our CBD sample. At first, the NMR spectrum shown in Figure A1 will be discussed.

The ${}^{1}$H-NMR of CBD can be divided into two characteristic parts. In the low ppm region from 0.9 ppm to 2.44 ppm a very characteristic set of multiplets can be observed. All of the peaks present in this spectrum are extremely common in all CBD samples and typically appear for all saturated C-H bonds in the molecular structure (see chemical structure in Figure A1). However, due to the high number of multiplets a detailed analysis of this region is obviously rather challenging. Moreover, since many other types of cannabinoids, which are the most common impurities [47], share a common carbon skeleton in this region; the presence of impurities could only be suggested and not confirmed by the analysis of this region. On the other hand, a second set of signals is observable starting from the multiplet at 3.14 ppm and ending with a rather broadened set of signals at 6.17 ppm. Here, all signals can clearly be assigned to CBD, because the molecular structure of all types of cannabinoids differs the most for this region. This is a very good example to prove that the CBD we use in our study is of very high purity are the signals at 4.56 ppm and 4.66 ppm. These signals appear exclusively for CBD, as it represents the *cis-trans* isomeric protons of the terminal ${=\mathrm{CH}}_{2}$-group, which are not present in any other cannabinoids. In addition, a broadened signal is measured at a shift of 6.26 ppm, which is due to a proton at C6’. This proves that neither CBDA (cannabidiolic acid) nor the narcotic $\Delta^{9}$-THC is present. Such observations were also made by Choi et al. [53]. Therefore, we expect our CBD to be of very high purity, since the most common impurities are not detected with NMR. The complete assignment of the characteristic signals can be found in Table A1.

To verify the results from the NMR investigation, we additionally measured ESI-MS (see Figure A2). The result also showed that the used CBD does not contain any significant impurities or traces of CBDA and $\Delta^{9}$-THC, respectively.

**Table A1:** ${}^{1}$H-NMR assignments for cannabidiol measured in CDCl${}_{3}$ at 500 MHz.

Position	${}^{1}$H-NMR
1	3.84 (1H, m)
2	5.57 (1H, s)
4	2.22 (2H, m), 2.09 (1H, m)
5	1.81 (2H, m)
6	2.40 (1H, m)
7	1.79 (3H, s)
9	4.56 (1H, m), 4.66 (1H, m)
10	1.65 (3H, s)
3’	6.27 (1H, *brs*)
5’	6.17 (1H, *brs*)
1”	2.44 (2H, *t*, ${}^{3}J_{HH}$ = 7.6 Hz)
2”	1.56 (2H, *dt*, ${}^{3}J_{HH}$ = 7.6 Hz, ${}^{1}J_{HH}$ = 15.2 Hz)
3”	1.29 (2H, m)
4”	1.29 (2H, m)
5”	0.88 (3H, ${}^{3}J_{HH}$ = 6.9 Hz)
2’OH	5.97 (1H, *brs*)
6’OH	4.60 (1H, *brs*)
